# Micro-level vulnerability assessment among agricultural communities of District Kupwara, Kashmir Himalaya

**DOI:** 10.1038/s41598-025-14950-9

**Published:** 2025-09-30

**Authors:** Kaneez Fatima, Masarat Jan, Ishfaq Ahmad, Majid Farooq, Mohammad Muslim

**Affiliations:** 1 Department of Environmental Science, University of Kashmir Hazratbal, Srinagar, J&K 190006 India; 2 Department of Ecology, Environment and Remote Sensing Government of Jammu and Kashmir, SDA Colony Bemina, Srinagar, J&K 190018 India

**Keywords:** Kashmir Himalaya, Inherent vulnerability, Vulnerability gap, Vulnerability severity, Ecology, Climate sciences, Ecology, Environmental sciences, Environmental social sciences

## Abstract

Livelihood and food security are pressing issues for mountain communities, particularly in the ecologically fragile and data-deficient regions of the Kashmir Himalaya. This study evaluates the inherent vulnerability of agricultural communities at the village level in Kupwara district using an indicator-based approach. A total of 356 villages were assessed using ecological and socioeconomic indicators of sensitivity, and adaptive capacity, collected from secondary sources. These indicators were standardized, weighted using Principal Component Analysis (PCA), and aggregated into composite indices for comparative analysis. Results show that 117 villages (33.14%) fall under high and 7 villages (1.96%) under very high vulnerability, while 211 villages are moderately vulnerable and over 90% of villages face moderate to high vulnerability. A comprehensive vulnerability gap and severity analysis was conducted, where the gap indicates the shortfall in adaptive capacity and severity measures potential damage. Village Hayihama emerged as highly sensitive, with a vulnerability gap of − 0.00893 and severity of 0.00312. The Kalrooch block had the highest severity, averaging a gap of − 0.00506 and severity of 0.00327. The research aims to guide policymakers by providing a robust framework to assess vulnerability, integrate risk perceptions into governance, and enhance resilience and adaptive capacity in farming communities.

## Introduction

Climate change poses a global challenge that profoundly impacts agriculture, a sector broadly recognized as being highly vulnerable to climatic fluctuations^1–3^. Numerous studies highlight how changes in temperature, precipitation patterns, and extreme weather events threaten food production and livelihoods, thereby necessitating targeted resilience measures^4–6^. The Himalayan region demonstrates these challenges acutely, owing to its ecological fragility and the critical dependence of local communities on rainfed agriculture^7,8^. Topographical constraints, erratic rainfall, and limited livelihood diversification further heighten these vulnerabilities, and urges for robust vulnerability assessments and a well-structured climate-adaptation plan^9,10^. Often a broad regional analysis overlooks micro-level variations in sensitivity, and adaptive capacity, reinforcing the importance of localized studies that can uncover specific drivers of vulnerability to tailor regional-specific adaptation^11–13^.

Agricultural systems, particularly in ecologically fragile regions, face substantial threats from rising temperatures, erratic rainfall, and extreme weather events, underscoring their pronounced vulnerability to climate change. For example, recent analyses have shown how shorter rainy seasons and warming trends disrupt the phenology and yields of both staple cereals and specialty crops, thereby exerting significant pressure on local food security and livelihoods^14,15^. Such stresses highlight the need to distinguish how climate hazards intersect with socio-economic conditions—an approach reflected in outcome vulnerability (the net effects of climate hazards) and contextual vulnerability (the pre-existing social, economic, and institutional factors that exacerbate or mitigate these effects). Moreover, inherent vulnerability integrates both dimensions, emphasizing innate social and ecological characteristics that predispose certain regions to greater risk. Recognizing these distinctions is crucial for formulating targeted adaptation strategies, particularly in mountainous areas like the Kashmir Himalaya, where steep terrain and infrastructural limitations amplify the consequences of climatic disruptions^16^.

The Kashmir Himalaya, the convergence of environmental, social, and political factors produces complex vulnerability patterns that differ significantly from one district to another. These variations arise from disparities in population density, literacy rates, economic reliance on agriculture, healthcare access, and the availability of natural resources such as water bodies and forests^17^. Researchers have increasingly highlighted the need for a dedicated Inherent Vulnerability Index tailored to the Kashmir context. Such an index would help capture the region’s unique challenges and guide policy interventions to better support at-risk communities^17^. Additionally, addressing climate change vulnerability in Kashmir demands integrated, multi-sectoral approaches that account for local governance structures, infrastructural gaps, and longstanding inequalities. In these contexts, the success of adaptation and resilience-building depends on empowering communities, promoting social justice, and ensuring inclusive, participatory decision-making^18^. However, climate change adaptation discourse has yet to achieve consistent emphasis on equity and justice, particularly with respect to marginalized communities in sensitive ecological regions^13^. This gap is especially evident in areas where socio-political complexities intersect with environmental risks, leading to uneven distribution of resources and differential impacts of climatic shocks on various demographic groups^19,20^.

District Kupwara in the northern Kashmir Valley illustrates many of these challenges, with steep slopes, fragmented landholdings, and a pronounced dependence on subsistence farming^17,21^. Limited livelihood diversification, suboptimal infrastructure, and minimal access to modern technologies magnify the community’s susceptibility to climate stressors^20,22^. Furthermore, the rugged terrain makes development of climate-resilient infrastructure, such as robust irrigation networks and efficient transportation systems, both logistically challenging and financially demanding^10,23^. Consequently, integrated strategies that consider both outcome vulnerability, to external hazards, and contextual vulnerability, shaped by internal social conditions, are needed to fully understand and address the district’s complex risk profile^24–26^.

An inherent vulnerability perspective focuses on the idea that vulnerability derives not merely from external climatic hazards but also from social, economic, and political structures^27–29^. Such a framework is particularly pertinent for Kupwara, where asymmetries in education, institutional support, and political representation significantly influence a community’s resilience^13,18^. In places where literacy rates and access to healthcare are relatively low, communities may struggle to adopt new farming practices or obtain timely climate information, thereby exacerbating the risks associated with unpredictable weather patterns^17,22^. Moreover, deeply rooted social hierarchies can impede collective action by restricting the agency of certain groups, such as women and smallholders, in local decision-making processes^19^. Recognizing these intra-community dynamics, an inherent vulnerability assessment provides a more nuanced lens through which to evaluate the interplay of structural inequalities and environmental change. This approach not only foregrounds localized risk factors but also highlights the importance of multi-level governance mechanisms that incorporate community voices in policy formulation^20,30^. This study addresses these challenges by conducting a micro-level vulnerability assessment of agricultural communities in Kupwara aimed to identify the key drivers and patterns of inherent vulnerability. While prior research in the Himalayan region has explored climate risks and adaptation^9,10,22^, this work distinguishes itself by adopting a micro-level lens specific to Kupwara, where resource constraints, inequitable governance, and ecological fragility converge. By emphasizing equity and justice in assessing vulnerability, the study provides a more nuanced depiction of how marginalization and limited agency amplify climate-related threats. It also stresses the role of local institutions—ranging from village councils to district-level administrative bodies—in facilitating or hindering adaptation measures^20,21^. In many Himalayan communities, decision-making power is concentrated among a few actors, and this can limit the inclusivity of adaptation programs, particularly for women, minorities, and landless laborers^19^. Engaging these groups in both the assessment and the solution design can foster greater ownership of adaptation strategies, thereby enhancing overall resilience^30,31^. By coupling these grassroots initiatives with policy interventions that address structural inequities—such as land tenure insecurity and uneven market access—stakeholders stand a better chance of forging meaningful, durable pathways toward resilience^13,19^.

This analysis goes beyond understanding vulnerability—it aims to equip communities and stakeholders with the tools to respond proactively to escalating climate challenges. In places like Kupwara, where environmental changes are outpacing adaptive measures, local voices must be at the forefront of shaping long-term strategies. As climate adaptation discourse increasingly emphasizes social justice and inclusivity^18,21^, micro-level assessments become essential for identifying where interventions can be most effective. By examining how factors such as educational disparities, land ownership, and governance structures intersect with climatic stressors, this study provides empirical insights to inform targeted policies and programs tailored to the realities of the Kashmir Himalaya.

## Study area

**Fig. 1:**
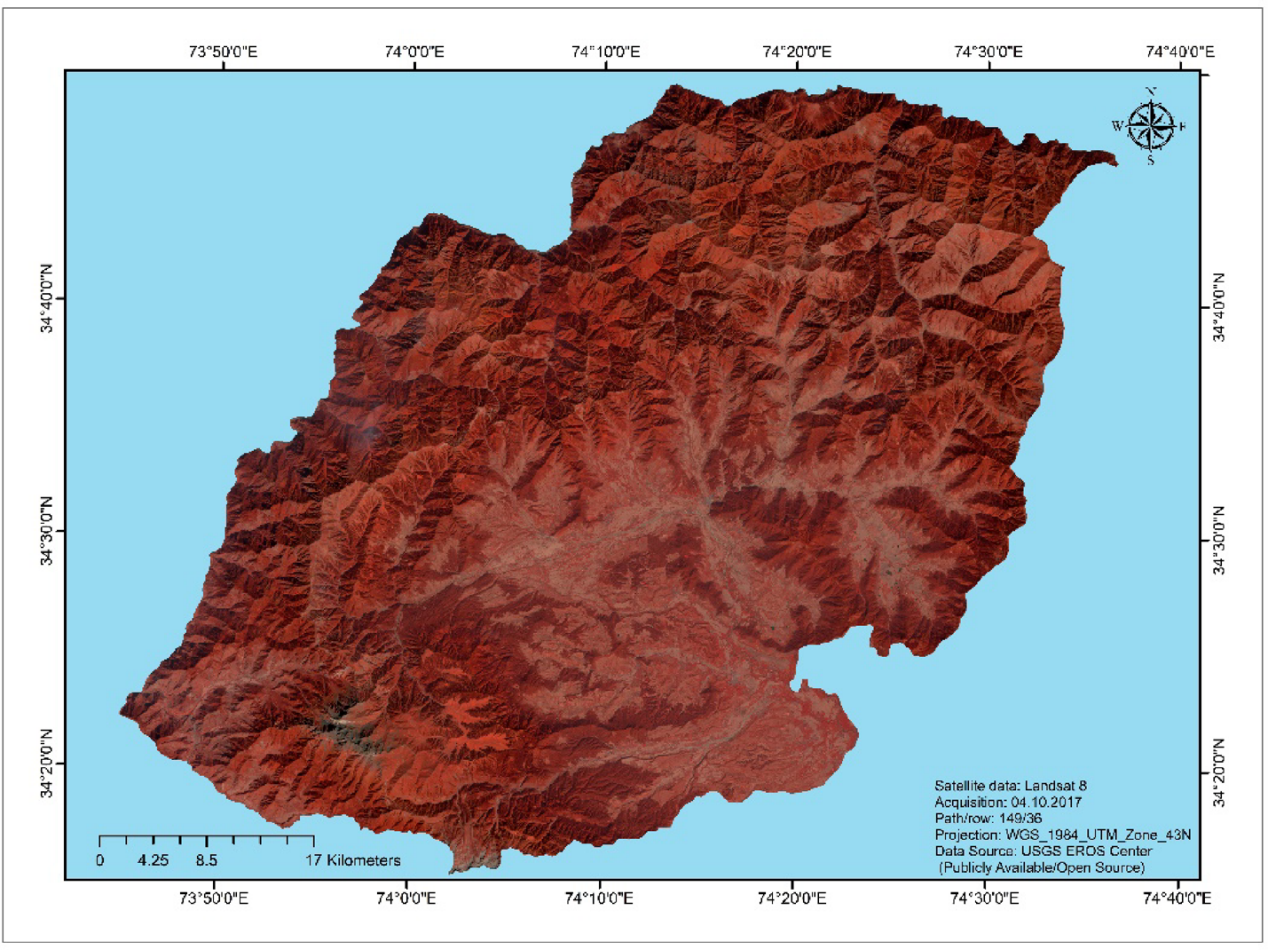
Study area map showing Landsat satellite imagery in a False Color Composite (FCC).

Kupwara district was initially part of the erstwhile Baramulla which was carved out as a separate district in July 1979. The district covers an area of 2379 square kilometers and boasts varying topography, with an average elevation of approximately 5300 feet above sea level. Positioned between 34.17 to 34.12 degrees North latitude and 73.10 to 73.16 degrees East longitude (Fig. 1), the district features hilly terrain in the western, northern, and eastern regions, while the southern areas are predominantly flat. Located in the northwest corner of the Kashmir Valley, Kupwara district is approximately 90 km away from Srinagar. It is bordered to the east and south by Baramulla district and to the west and north by the Line of Actual Control (L.O.C). In Kupwara, as in the rest of the state, agriculture serves as the primary livelihood source.

Kupwara district is subdivided into 3 Tehsils, 11 Rural Development blocks, and comprises 356 inhabited villages. As per the 2011 census^32^, the district had a population of 870,354, consisting of 474,190 males and 396,164 females. This represents an approximate increase of 33.85% compared to the 2001 census and constitutes 14.88% of the total population of the state. The majority of the population resides in rural areas, with only 104,000 individuals living in urban areas according to the 2011 census^32^. Among the district’s total population, Schedule Castes make up around 0.01%, while Schedule Tribes account for 8.08%. Kupwara boasts an average literacy rate of 64.51%, surpassing the national average of 59.5%. Male literacy stands at 64%, while female literacy is at 41%.

## Methodology

**Fig. 2:**
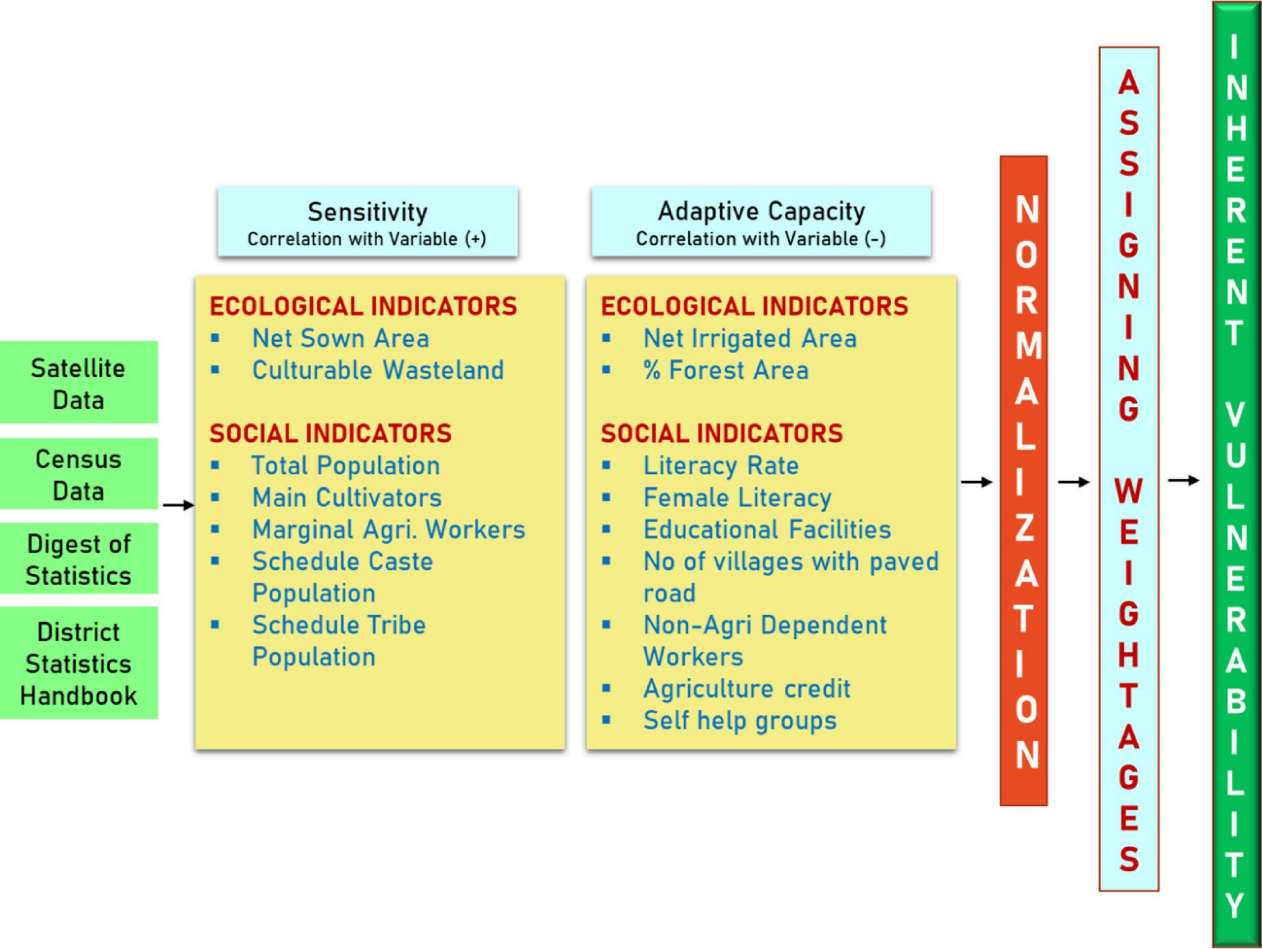
Flow chart of the overall methodology.

Inherent vulnerability assessments disaggregate overall vulnerability into its core components, sensitivity and adaptive capacity crucial for understanding the underlying drivers of risk. This approach is consistent with widely accepted frameworks, such as those proposed by the IPCC, which conceptualize vulnerability as a function of sensitivity and adaptive capacity. In this study, a structured, indicator-based methodology is employed to assess inherent vulnerability, climate-related vulnerability, and associated risks faced by farming communities at the village level. The framework integrates several analytical techniques, including indicator selection, normalization, weighting, composite index computation, vulnerability classification, gap and severity analysis, and spatial mapping, to ensure a comprehensive and data-driven assessment^33^. An overview of the methodological approach is provided in Fig. 2, with further details outlined below.

## Selection of indicators

Based on a comprehensive review of the literature and available secondary data sets, theoretically important and policy-relevant socio-economic variables were selected under major components of demographic profile, socio-economic status, livelihood activity, human resource capacity, economic security, infrastructure, basic facilities and agricultural livelihood strategies^33^. The selection of indicators was guided by a well-defined vulnerability conceptual framework and expert validation processes to ensure relevance, accuracy, and applicability.

*Village selection* A total of 356 villages in the study region (excluding uninhabited and forest villages) were considered for analysis. Some villages and blocks had missing data for certain indicators, which may have influenced the vulnerability assessment. These gaps were addressed using an imputation method or, in some cases, by excluding incomplete data from the analysis.

*Indicator identification* A preliminary set of indicators was compiled based on existing literature and frameworks on climate vulnerability.

*Expert validation* The proposed indicators were reviewed through structured interactions with a diverse group of experts, including academic researchers, policymakers, local agricultural officers, and climate scientists. The validation process included structured interviews, the Delphi method, focus group discussions, and surveys.

*Indicator refinement* Experts evaluated the indicators based on their relevance, consistency, and data availability. The final selection of indicators was refined based on expert feedback and data availability at the village level.

The finalized set of indicators included 15 variables, categorized as follows:

*Sensitivity indicators (6)* These reflect the degree to which communities are affected by external shocks. They exhibit a positive correlation with vulnerability (Table 1).

**Table 1:** Indicators selected for sensitivity component.

Dimensions	Indicators	Explanation	Correlation with vulnerability	Data source	References
Ecological dimension	% of Net sown area in (ha)	A higher proportion of cultivated land indicates more significant exposure to environmental risks (e.g., drought, pests), thereby increasing overall sensitivity to climate variability. This is chosen to reflect how intensively the land is used and how many livelihoods depend on potentially vulnerable farmland	+	^32,34^	^1,35,36^
	% of Cultivable waste land in (ha)	Cultivable wasteland refers to land fit for cultivation but left uncultivated for at least five years. A larger share of such land can signal abandonment or degradation. It reflects a higher risk because these lands may be prone to adverse conditions (e.g., erosion), exacerbating vulnerability	+	^32,34^	^1,37^
Social dimension	Total population per unit of agriculture area	A higher population relative to available agricultural land intensifies pressure on resources, leading to overexploitation and land degradation. This indicator shows how demographic pressure elevates vulnerability by stressing limited agricultural land	+	^32,38^	^1,39,40^
	% of ST/SC population	Scheduled Tribes (ST) and Scheduled Castes (SC) often face socioeconomic marginalization and rely heavily on natural resources. A larger ST/SC population typically indicates higher sensitivity to resource scarcity, as these communities may have limited adaptive capacity and fewer livelihood options	+	^32,38^	^41^
	% of Main cultivators of total population	Households whose primary occupation is cultivation may have better resource access (land ownership) but face elevated income risk if crop failures occur. Greater involvement in farming (> 6 months) signifies higher sensitivity due to reliance on potentially vulnerable agricultural systems	+	^32,38^	^1,40,42^
	% of total marginalized agriculture workers	Marginal workers do not own land and depend on daily wages. An increase in their share indicates a larger at-risk group with fewer livelihood alternatives, thereby raising overall susceptibility to shocks (e.g., crop failure, market fluctuations)	+	^32,38^	^1^

*Adaptive capacity indicators (9)* These represent the ability of farming communities to cope with or adapt to climate stressors. They exhibit a negative correlation with vulnerability (Table 2). Adaptive capacity in the assessment moderates risk perception and influences strategic decisions, but it must be balanced with realistic evaluations of sensitivity to avoid false scrutiny^43^.

**Table 2:** Indicators selected for Adaptive capacity component.

Dimensions	Indicators	Explanation	Correlation with Vulnerability	Data Source	Reference
Ecological dimensions	% of net irrigated area in (ha)	Irrigation reduces dependence on erratic rainfall, thereby enhancing agricultural productivity and livelihood security. A higher percentage of irrigated area typically **lowers** vulnerability by stabilizing crop production	**–**	^32,34,38^	^44,45^
	% of total forest area	Forest cover helps maintain soil fertility, prevent erosion, and regulate hydrological cycles. An increased forest area generally **reduces** vulnerability by protecting agricultural lands and water resources from degradation	**–**	^34,38^	^46,47^
Social dimensions	Literacy rate (% of literate population)	Literacy is a key driver of adaptive capacity, enabling better access to information, alternative livelihoods, and social services. A higher literacy rate **lowers** vulnerability by improving households’ resilience to shocks	**–**	^32^	^48,49^
	Sex ratio (female per 1000 male)	A balanced or higher female-to-male ratio often indicates greater women’s participation in farming and decision-making. Enhanced gender equity can **reduce** vulnerability by diversifying labor and improving household resilience	**–**	^32^	^50^
	Non agriculture dependent workers	A higher number of individuals with non-farm skills and income sources reflects economic diversification. This **lowers** vulnerability by reducing sole reliance on agriculture and spreading risk across multiple livelihood activities	**–**	^32,38^	^1,47^
	Availability of education facilities	Improved access to education facilities enhances skill development, raises awareness, and fosters innovation in farming practices. This generally **decreases** vulnerability by building human capital and adaptive capacity	**–**	^32,38^	^51,52^
	Availability of roads	Better road connectivity provides market access, lowers transportation costs, and creates opportunities for off-farm employment. This **decreases** vulnerability by improving economic options and resource mobility	**–**	^32,34,38^	^48,49^
	Agriculture credit societies	Availability of credit enables farmers to invest in improved seeds, technology, and other resources crucial during critical periods. This **lowers** vulnerability by providing financial security and reducing the impact of climate-induced crop failures	**–**	^38^	^45^
	Self-help groups	Self-help groups offer financial, social, and technical support, often involving women’s participation in farming. Increased group participation **reduces** vulnerability by strengthening social networks and resource-sharing mechanisms	**–**	^38^	^1^

A significant portion of the demographic data was sourced from the 2011 Census of India, along with additional data from relevant government and institutional reports.

## Normalization of indicators

Building on the core vulnerability dimensions, sensitivity and adaptive capacity—defined above, this section explains the quantitative steps by which each indicator is normalized, weighted via PCA, and aggregated. Since the selected indicators had different units and measurement scales, a standard normalization process was applied to ensure comparability. The linear minimum–maximum scaling method was employed, a widely used approach in hierarchical vulnerability assessments^53,54^.

Normalization for Sensitivity Indicators (positively correlated with vulnerability):

$${\text{Normalised}}\;{\text{value}}\left( {\text{N}} \right) = \frac{{{\text{Actual}}\;{\text{Value}} - {\text{Minimum}}\;{\text{value}}}}{{{\text{Maximum}}\;{\text{value}}{-}{\text{Minimum}}\;{\text{value}}}}$$

Normalization for Adaptive Capacity Indicators (negatively correlated with vulnerability):

$${\text{Normalized}}\;{\text{valve}}\left( {\text{N}} \right) = \frac{{{\text{Maximum}}\;{\text{Value}} - {\text{Actual}}\;{\text{Value}}}}{{{\text{Maximum}}\;{\text{Value}} - {\text{Minimum}}\;{\text{Value}}}}$$

This process ensured that all indicators were converted into a dimensionless scale ranging from 0 to 1, facilitating direct comparisons and aggregation into composite indices.

## Summarizing and weighting of indicators

After all indicators were rescaled to the common 0–1 interval using min–max normalization, we organized the data into two separate matrices for adaptive capacity, each with rows representing villages and columns representing their respective indicators. For each matrix we calculated the Pearson correlation matrix, rather than the covariance matrix, so that variables measured on different original scales would contribute equally to the subsequent analysis. Solving the eigenvalue–eigenvector system of this correlation matrix decomposed the total variance into orthogonal principal components (PCs). We inspected the eigenvalue spectrum (scree plot). We applied the Kaiser criterion, retaining only the first component in each case because its eigenvalue exceeded unity, and it alone captured more than 70% of the variance, well above the conventional threshold for interpretability in socio-environmental studies. The component loadings (the elements of the retained eigenvector, scaled by the square root of its eigenvalue) quantify the marginal contribution of each indicator to the principal component; we converted these loadings into objective weights by taking their absolute values, thereby ignoring sign while preserving magnitude, and normalising them so that their sum equals one^55^. This transformation maintains comparative influence across indicators and facilitates direct use in a weighted-sum index. To guard against redundancy, we then computed pairwise correlations among the original indicators; whenever two variables exhibited a correlation coefficient with absolute value greater than 0.90, we either averaged them into a composite indicator (if both captured complementary aspects of the same phenomenon) or, when conceptually duplicative, dropped the one with the lower communality. These steps ensured that the final weighting scheme was data-driven, statistically sound, and free from spurious inflation of highly collinear variables.

## Computation of total vulnerability

Vulnerability describes the inherent traits of a system or community that predispose it to harm from climate‐related hazards, regardless of the frequency or intensity of specific events. It arises from a complex interplay of social, economic, environmental and institutional conditions such as poverty, health status, educational attainment, governance quality and resource access that together shape how severely a hazard will affect that system. In line with the IPCC Fifth Assessment Report (AR5), vulnerability is understood as the propensity to be adversely affected, incorporating both the sensitivity to damage and the capacity to cope and adapt. Accordingly, we conceptualize vulnerability (V) as a function of sensitivity (S) and adaptive capacity (AC), with the latter acting to reduce overall vulnerability. To capture this inverse relationship, we first normalize AC to a 0–1 scale and then compute its complement, (1–AC), which grows larger only when adaptive capacity is low. Finally, we aggregate sensitivity and inverted adaptive capacity to obtain the composite vulnerability index:

$${\text{V}} = {\text{S}} + \left( {{1} - {\text{AC}}} \right)$$

where V = Total vulnerability, S = Sensitivity score (positively contributing to vulnerability), AC = Adaptive capacity score (negatively contributing to vulnerability), This formulation enabled a systematic evaluation of the overall vulnerability of farming communities, incorporating resilience-related factors.

## Vulnerability categorization

To facilitate meaningful interpretation, vulnerability levels were classified based on percentile rankings:

*Low vulnerability* Below the 33rd percentile.

*Medium vulnerability* Between 34 and 66th percentiles.

*High vulnerability* Above the 66th percentile.

Additionally, villages were further categorized into distinct vulnerability levels, following established methodologies^56^. This classification approach allows for targeted interventions based on vulnerability^1,57^.

## Vulnerability gap and severity analysis

To evaluate the degree to which farming communities deviate from an acceptable vulnerability threshold, two advanced metrics were computed. The Vulnerability Gap (V1) and Vulnerability Severity Index (V2), as proposed by Adger, are metrics designed to assess the extent and depth of vulnerability within communities, particularly in the context of environmental and social challenges. While both metrics use the same base formula and normalization approach, V1 applies a linear deviation, whereas V2 applies a squared deviation. This mathematical difference means that V2 places greater emphasis on larger deviations from the threshold, thus highlighting communities that are significantly worse off. These generalized measures of vulnerability, proposed by Adger^58^, assess both the severity of vulnerability and the distribution of risk within vulnerable communities.

*Vulnerability gap* The Vulnerability Gap quantifies the extent to which a community’s vulnerability exceeds a defined threshold:

$${\text{V}}_{{1}} = \frac{1}{n}\left[ {\mathop \sum \limits_{i = 1}^{q} \left( {w_{0} - \frac{wi}{{w0}}} \right)^{1} } \right]$$

*Vulnerability severity* The Vulnerability Severity Index accounts for the depth of vulnerability, capturing the degree of risk distribution within communities:

$${\text{V}}_{{2}} = \frac{1}{n}\left[ {\mathop \sum \limits_{i = 1}^{q} \left( {w_{0} - \frac{wi}{{w0}}} \right)^{2} } \right]$$

where w_i_ = the wellbeing of individual i (individual, community, village etc.), w_0_ = threshold level of well-being representing danger or vulnerability, n = total number of individuals, q = number of individuals above the vulnerability threshold.

In risk assessments, setting w_0_ as the maximum observed value max(wi) ensures that all deviations are normalized relative to the worst-case scenario. In this analysis, we set w0 = 0.6 using the maximum observed well-being representing danger or vulnerability, to normalize all deviations relative to the best-case scenario, thereby scaling the vulnerability measures between 0 and 1. Normalizing by max(wi) scales all values between 0 and 1, making comparisons more intuitive. This analysis provides a nuanced understanding of which communities are at the highest risk and by what margin.

## Vulnerability mapping

To effectively visualize and communicate the findings, non-spatial data comprising sensitivity, adaptive capacity, and overall vulnerability scores were integrated with spatial village boundary layers using ArcGIS Desktop 10.8.1 (licensed under the ESRI EIGAP program). The methodology involved collecting village-level indicators for sensitivity, and adaptive capacity, followed by normalization and weighting through Principal Component Analysis (PCA) to compute composite vulnerability scores. These scores were then linked to spatial data using a common identifier, such as village name or code. The integrated data were visualized in GIS by classifying vulnerability levels into categories (e.g., very low to very high) based on percentile rankings, and represented through appropriate color symbology. The resulting high-resolution vulnerability maps offer a spatial understanding of risk distribution, supporting policymakers in prioritizing interventions and designing resilience strategies tailored to specific village contexts.

## Result

**Fig. 3:**
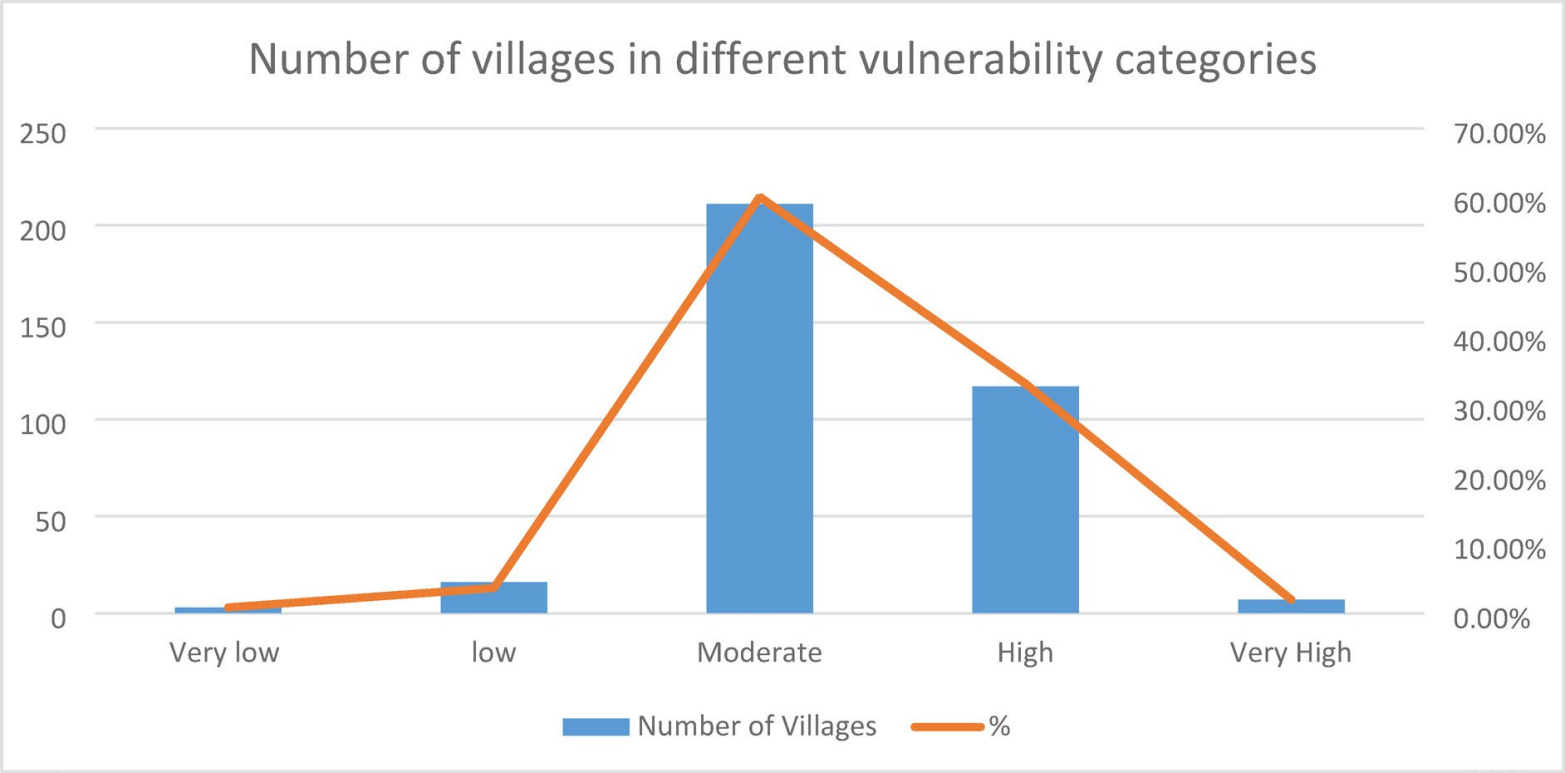
Number of villages fall under different vulnerability categories.

**Fig. 4:**
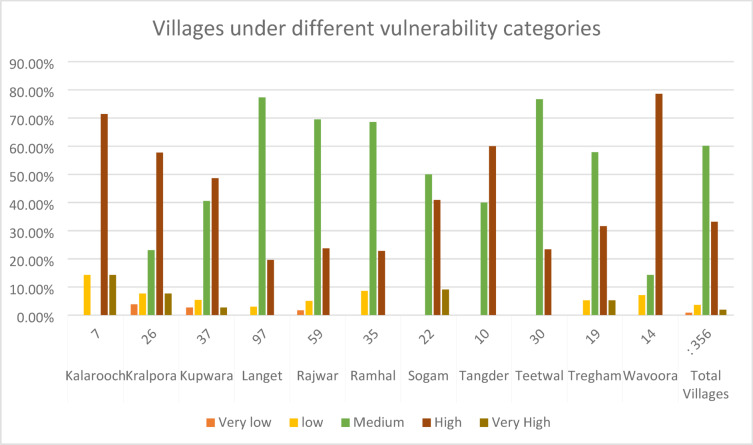
Block wise vulnerability categories of villages of district Kupwara.

**Fig. 5:**
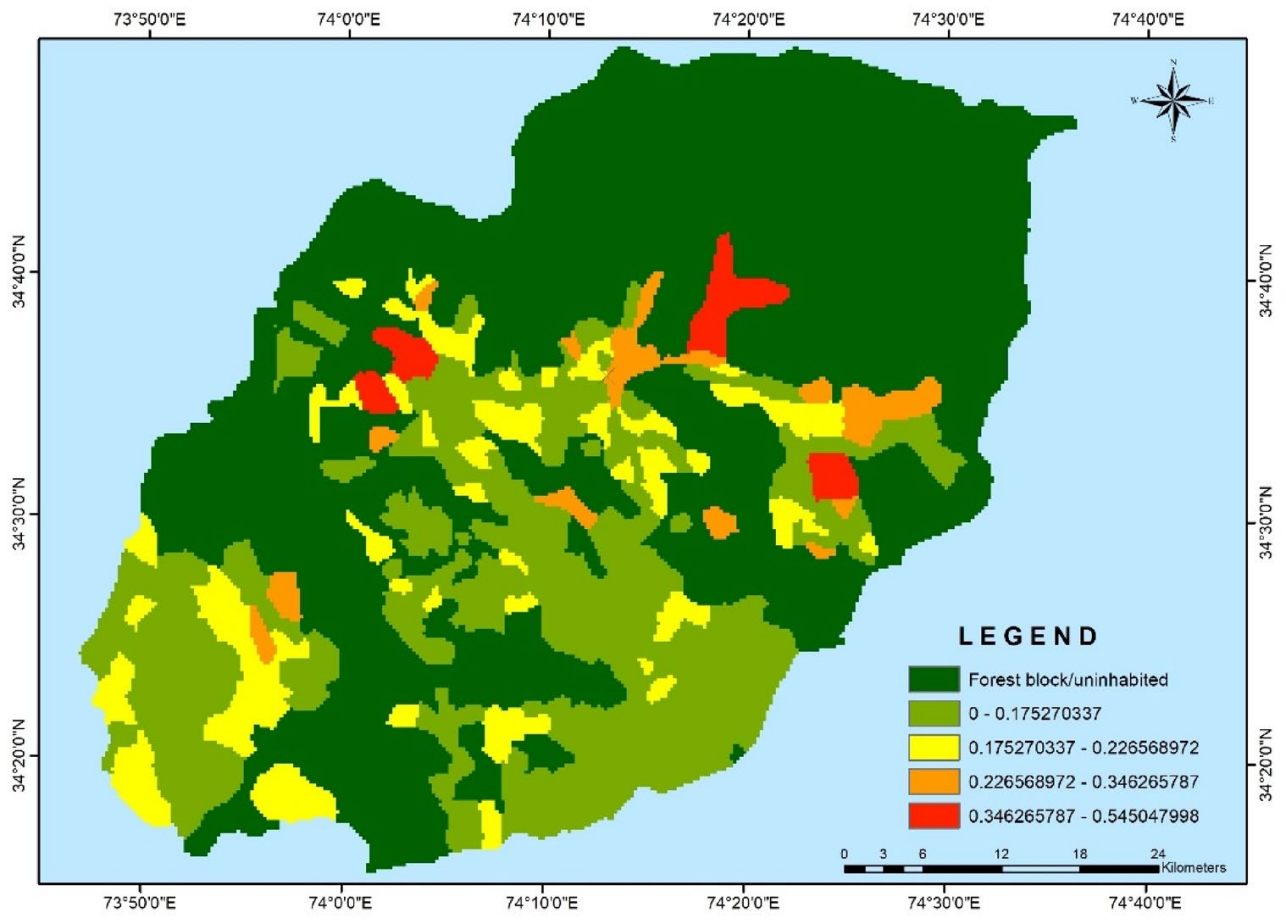
Map depicting the distribution of Sensitivity across the Kupwara district.

**Fig. 6:**
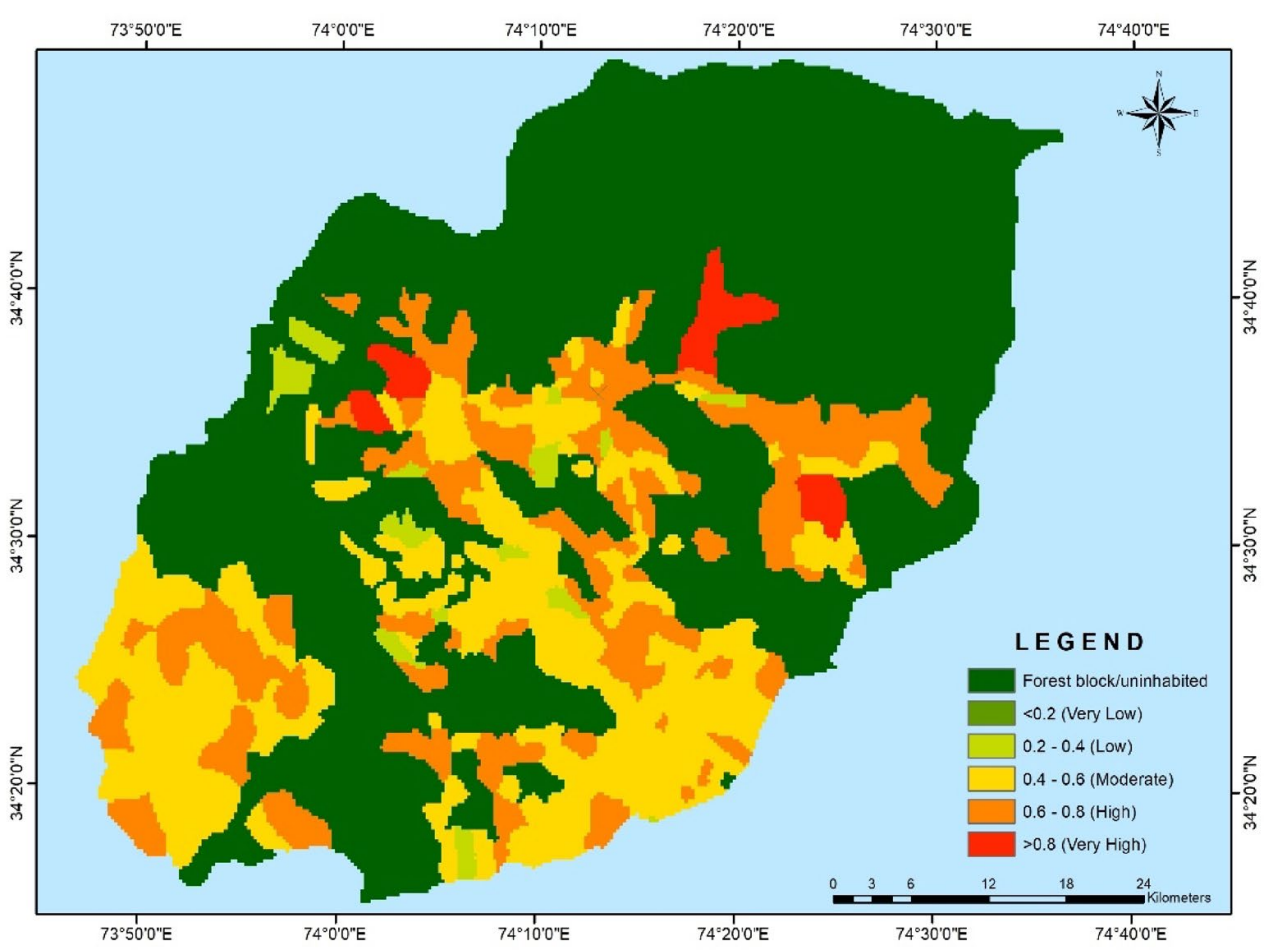
Map depicting the distribution of Adaptive capacity across the Kupwara district.

**Fig. 7:**
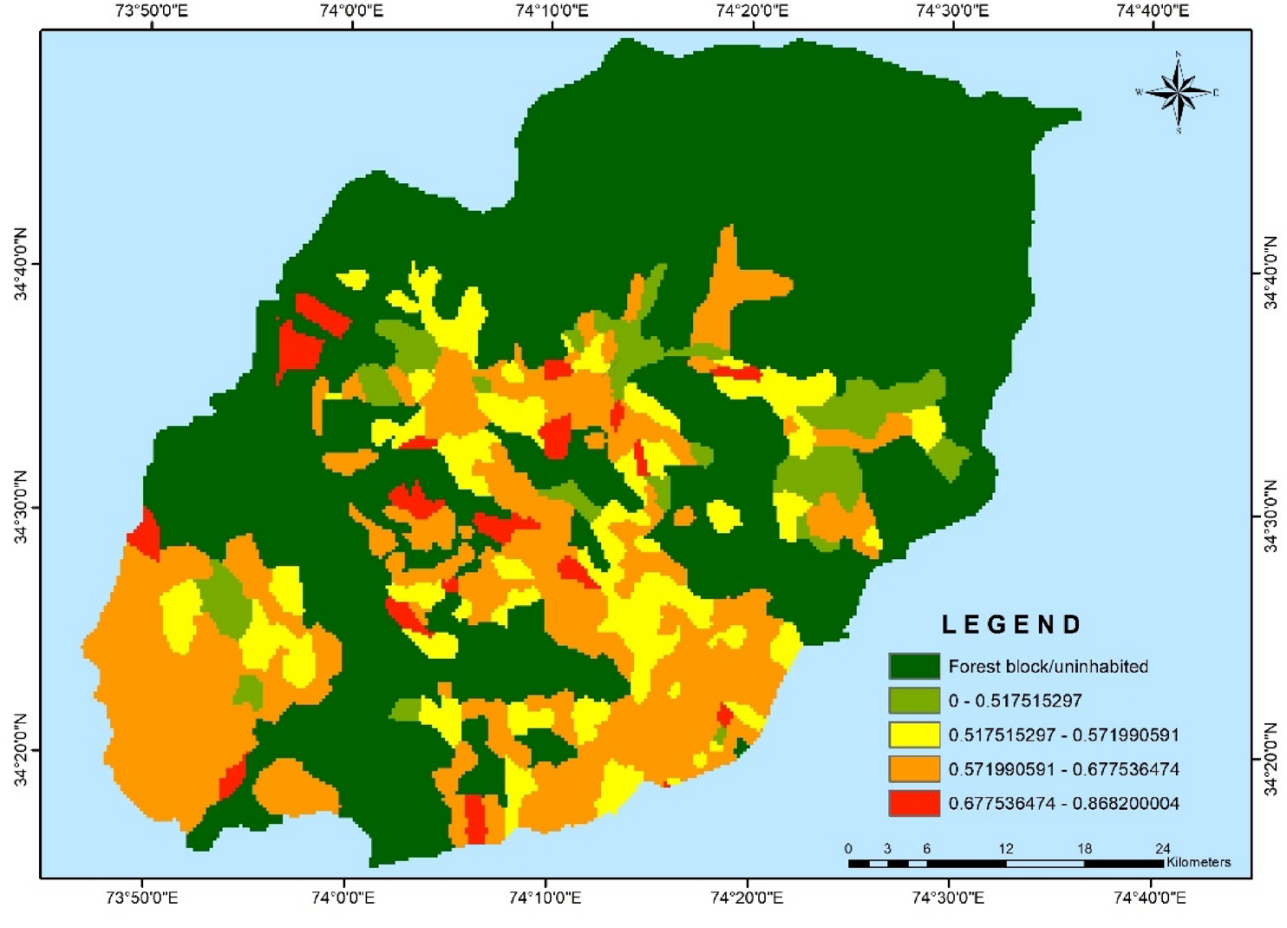
Map depicting the Vulnerability across the Kupwara district.

The village-wise vulnerability indices of Kupwara have been executed for different blocks for socio-demographic and common property resource indicators. Out of the 15 selected indicators 9 were used to access the adaptive capacity (Table 1) and 6 indicators were used to determine the sensitivity of villages of district Kupwara (Table 2). The selected indicators were normalized and weighted by applying PCA method. The analysis of vulnerability distribution across different blocks in Kupwara district revealed significant variations. Only three villages, each from Kralpora (3.84%), Kupwara (2.70%), and Rajwar (1.69%) blocks, were categorized as having very low vulnerability (> 0.2). Surprisingly, the other eight blocks had no villages falling under this category. A total of 16 villages (3.65%) were classified as having low vulnerability (0.2–0.4), excluding villages from Sogam, Tangder, and Teethwal blocks (Fig. 3). The moderate vulnerability category (0.4–0.6) exhibited the highest number of villages, with 10 out of 11 blocks showing vulnerability. These blocks, namely Langate (77.31%), Teethwal (76.66%), Rajwar (69.49%), Ramhal (68.57%), Trehgam (57.84%), Sogam (50.0%), Kupwara (40.54%), Tregham (40.0%), Kralpora (23.07%), and Wavoora (14.28%), contributed to this category.The high vulnerability class (0.6–0.8) included villages from block Wavoora (78.57%), Kalrooch (71.22%), Tangder (60%), Kralpora (57.69%), Kupwara (48.64%), Sogam (40.90%), Tehgam (31.57%), Teetwal (23.33%), Rajwal (23.72%), Ramhal (22.80%), and Langate (19.58%). The very high vulnerability (> 0.8) category comprised villages from block Kalrooch (14.28%), Sogam (9.09%), Kralpora (7.69%), Tregham (5.26%), and Kupwara (2.70%) in (Fig. 4 and Table 3). These variations in vulnerability indices among the villages of different blocks can be attributed to various factors, including heavy reliance on agriculture, as well as ecological and socioeconomic elements such as forest cover, literacy rate, educational facilities, and road infrastructure in and around the villages and the respective blocks in Kupwara district. From the two components of vulnerability (sensitivity and adaptive capacity) in (Figs. 5 and 6) the inherent vulnerability of all the villages was calculated and sorted on block and village level as very low, low, medium–high, and very high accordingly (Table 4). Among the 362 villages in district Kupwara, the majority, 356 villages (98.3% of the area), are inhabited, while the remaining (1.7%) comprised uninhabited villages and forest blocks. Within the 356 inhabited villages, approximately 7 villages (1.96%) were found to be having very high vulnerability (> 0.8). Additionally, about 118 villages (33.14%) fell under the category of high vulnerability (0.6–0.8), 212 villages (60.11%) were moderately vulnerable (0.4 and 0.6), 16 villages (3.65%) were found to have low vulnerability (0.2 and 0.4), and 3 villages (0.84%) were identified as having very low vulnerability (> 0.2) in (Fig. 7).

**Table 3:** Block-wise vulnerability categories of villages of district Kupwara.

S. No.	Name of Blocks	Number of villages in each block			Block vise percentage of vulnerability categories						
			Very Low (> 0.2) (%)	Low (0.2–0.4) (%)	Moderate (0.4–0.6) (%)	High (0.6–0.8) (%)	Very High (< 0.8) (%)	AverageGap	SD	Average Severity	SD
1	Kalarooch	7	0.00	14.28	00.00	71.42	14.28	− 0.00506	0.0005	0.00327	0.0007
2	Kralpora	26	3.84	07.69	23.07	57.69	07.69	− 0.00459	0.0004	0.0027	0.0006
3	Kupwara	37	2.70	05.40	40.54	48.64	02.70	− 0.00442	0.0004	0.00248	0.0006
4	Langate	97	0.00	03.00	77.31	19.58	00.00	− 0.00363	0.0001	0.00157	0.0001
5	Rajwar	59	1.69	05.08	69.49	23.72	00.00	− 0.00389	0.0001	0.00184	0.0001
6	Ramhal	35	0.00	08.57	68.57	22.80	00.00	− 0.00408	0.0001	0.00203	0.0001
7	Sogam	22	0.00	00.00	50.00	40.90	09.09	− 0.0048	0.0003	0.0029	0.0005
8	Tangder	10	0.00	00.00	40.00	60.00	00.00	− 0.00436	0.0002	0.0023	0.0002
9	Teetwal	30	0.00	00.00	76.66	23.33	00.00	− 0.00367	0.0007	0.0016	0.0001
10	Tregham	19	0.00	05.26	57.84	31.57	05.26	− 0.00481	0.0005	0.00303	0.0008
11	Wavoora	14	0.00	07.14	14.28	78.57	00.00	− 0.00453	0.0002	0.00249	0.0008
Total villages		356	0.84	03.65	60.11	33.14	01.96	− 0.00453	0.0003	0.00238	0.0005

**Table 4:** Number of village in different vulnerability categories.

	Number of Village in different Vulnerability Categories				
Categories	Very low	low	Moderate	High	Very High
Number of Villages	3	16	212	118	7
% Area	0.84%	3.65%	60.11%	33.14%	1.96%

**Fig. 8:**
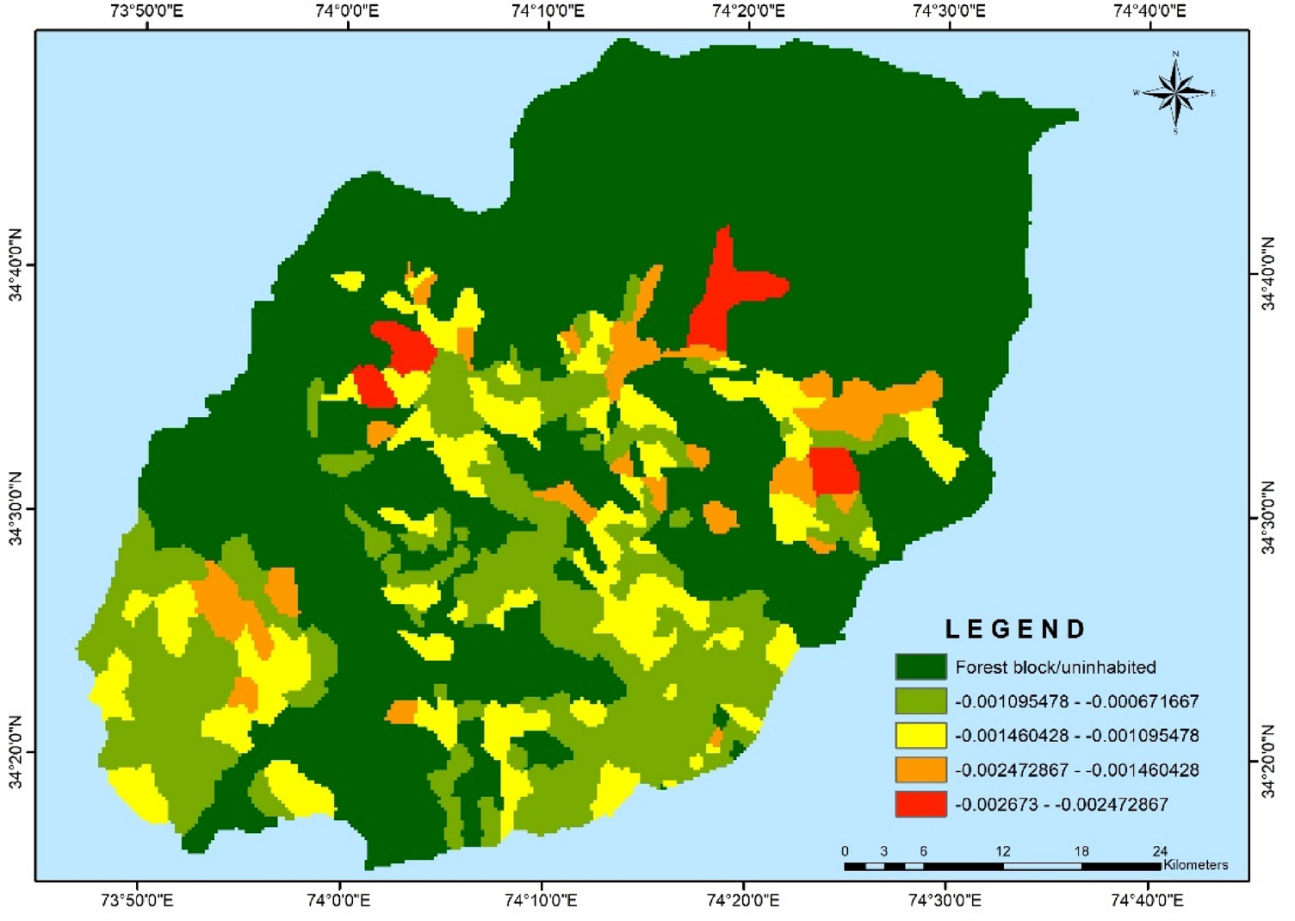
Map depicting Gap in vulnerability across the Kupwara district.

**Fig. 9:**
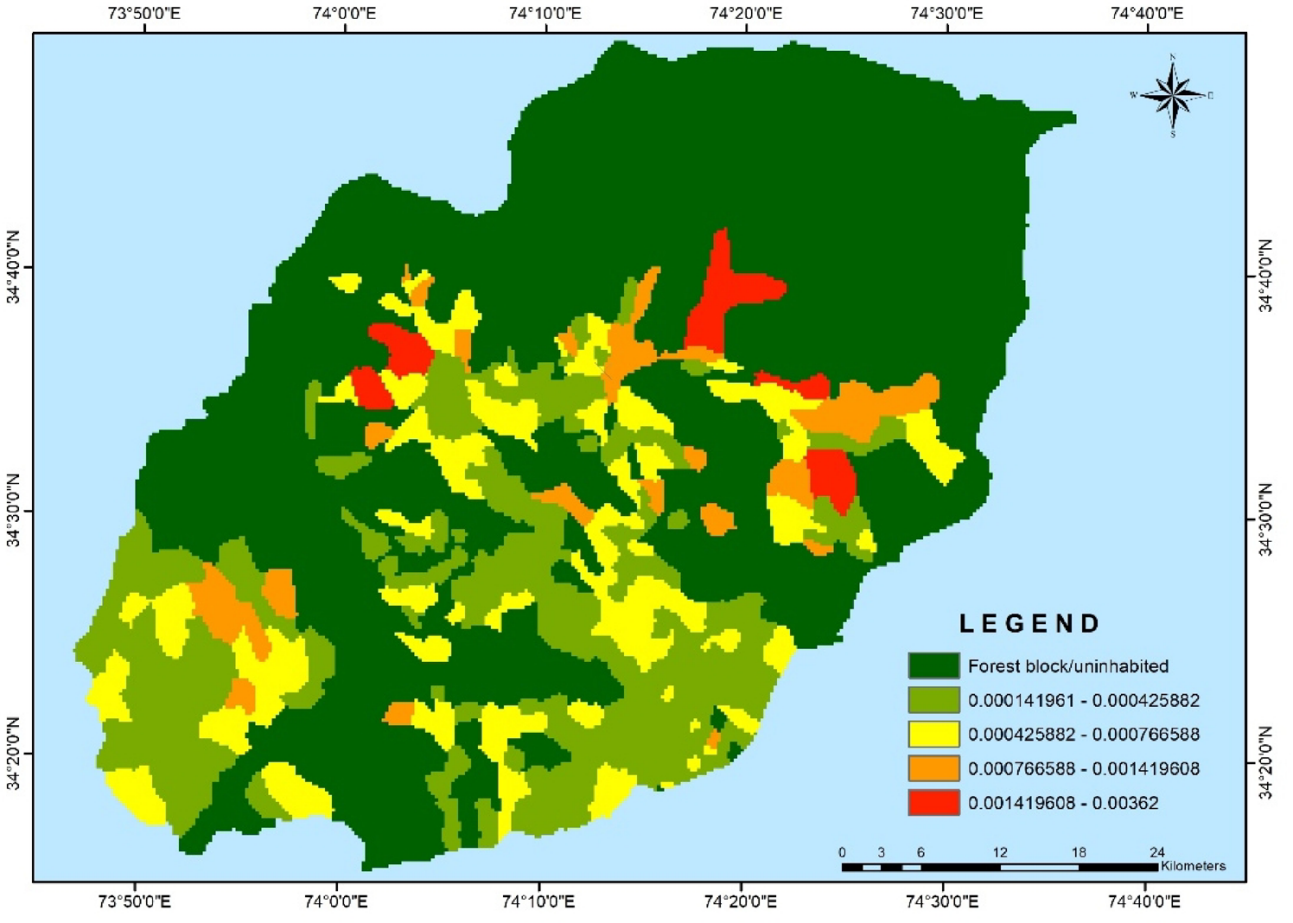
Map depicting Severity in vulnerability across the Kupwara district.

At the block level, Kalrooch block showed the highest average gap and severity values of − 0.00506 and 0.00327, respectively (Table 3). This was primarily because this block had the highest number of villages above the vulnerability threshold (categorized as high and very high vulnerability). Conversely, Langate block had the lowest vulnerability gap and severity values of − 0.00363 and 0.00157, respectively, with most of its villages falling into the moderately and low vulnerability categories (Figs. 8 and 9). The vulnerability gap and severity within the vulnerable villages exhibited a consistent pattern. At the village level, Hayihama in Kupwara block had a gap value of − 0.00893 and a severity value of 0.00941. On the other hand, Bata Pora in Langate block had the lowest risk of becoming more vulnerable, with gap and severity values of − 0.00317 and 0.00119, respectively.

## Discussion

The overall high inherent vulnerability observed at the village level in the Kupwara district, with 7 villages categorized as very high vulnerability, 118 as high vulnerability, and 212 as moderate vulnerability, can be directly attributed to several factors. Among these are the lack of education facilities, low literacy rates, inadequate road infrastructure, limited access to agricultural credit societies, and insufficient support from self-help groups in the villages of the district. Moreover, the uneven vulnerability encountered by agricultural communities in mountainous regions is influenced by a range of factors. These factors encompass the absence of effective skills development programs^59,60^, insufficient infrastructure facilities^61,62^, limited financial capacities, and the absence of localized credit systems like self-help groups and marketing societies^63–65^. These factors collectively contribute to the heightened vulnerability of the communities in the district, highlighting the need for targeted interventions and comprehensive support systems to enhance resilience and reduce vulnerability among these regions.

The combined forest area of all villages in the district covered a significant area, accounting for a small percentage of the total land area. On the other hand, the total irrigation area spanned a much smaller portion of the district’s total area. These observations suggest a lower ecological adaptive capacity, likely due to intensified agriculture in certain villages with minimal forest cover, indicating an imbalance in agricultural systems^66^. Further, Himalayan ecosystems are highly vulnerable due to the stress caused by forest land diversion, increasing pressure from human population, exploitation of natural resources, infrastructure development, mining, and other related challenges. The effect of these current stressors is likely to be exacerbated due to climatic changes, which would be additional^67^. This reduced adaptive capacity could be attributed to the degradation of forest resources and unirrigated cropland^68,69^. Other contributing factors include water scarcity^62^, changing climatic patterns^59^, and a loss in agrobiodiversity^70^. These factors are expected to exacerbate the situation and render the communities even more vulnerable. Enhancing adaptive capacity and mitigating inherent vulnerability is of paramount importance. Prior research underscores the significance of forest conservation in preserving stability and upholding the diversity and productivity of agricultural systems in mountainous regions^3,71^. A comparative analysis with agricultural communities in Central Himalayas^72^, Western Himalayas^73,74^, and Himachal Pradesh^75^ sharing similar agricultural practices reveals that resilience-building measures have been proposed using vulnerability assessments. These strategies align closely with the findings and recommendations of our research, highlighting the importance of targeted interventions to enhance adaptive capacity and reduce inherent vulnerability in mountainous agricultural systems. The high inherent vulnerability is a consequence of the communities’ heavy reliance on fragile agriculture systems. The significant vulnerability of agricultural communities in the region stems from their heavy dependence on fragile agricultural systems. Our study reveals that across 356 villages, approximately 139,907 individuals accounting for 16.07% of the total population—are engaged as marginal agriculturists and main cultivators. This highlights an urgent need to enhance infrastructure, diversify livelihoods, and establish strong institutional networks, all of which are essential for strengthening adaptive capacity and ensuring long-term resilience. Several critical factors influence the vulnerability of these agricultural communities, including access to irrigation, communication networks, village remoteness, institutional support, and natural resource availability. Addressing these challenges through targeted interventions has been widely advocated in scientific literature. Ensor^59^ emphasizes the importance of community-based adaptation approaches that incorporate social capital and institutional support to build resilience in rural agricultural regions. Adger^76^ stress the role of adaptive governance and decentralized decision-making in reducing vulnerability and enhancing coping mechanisms. The effective adaptation relies on a mix of technological advancements, improved market access, and localized capacity-building initiatives to mitigate long-term risks^77^. Given these insights, it is imperative to adopt a comprehensive adaptation strategy that integrates both social and biophysical dimensions. A holistic approach will allow for more effective planning, enabling vulnerable agricultural communities to better navigate climatic and socioeconomic changes while fostering sustainable resilience.

The villages in all blocks of the district are increasingly becoming more vulnerable due to the lack of various facilities, such as educational opportunities, market access, and infrastructure. Glwadys^78^ also emphasized the need to address issues like higher rural populations, unemployment, low literacy rates, and limited infrastructure to reduce the sensitivity of rural communities. To reduce agricultural vulnerability in the region, it is essential to tackle these components of vulnerability^66^ as doing so can effectively reduce disaster risks. The Vulnerability Index, derived from these components, ranks villages and blocks within the district, guiding development planning interventions. It becomes evident that addressing the inherent sensitivity of the agricultural sector is crucial in reducing vulnerability. This can be achieved by empowering small and marginal farmers through improved access to education and healthcare^3^. Providing skill-building trainings is also essential to enable them to access available technology, markets, and infrastructure to enhance farm production sustainability. Special attention should be given to backward villages to reduce the vulnerability of the agricultural sector to potential climate change risks. To strengthen community resilience, targeted training programs on climate-resilient farming, including drought-resistant crops, water management, and soil conservation, should be implemented. Farmer cooperatives and self-help groups can enhance market access, bargaining power, and knowledge-sharing. Expanding extension services and integrating digital tools, such as mobile advisory platforms, will enable informed decision-making on weather patterns, pest control, and crop planning. Additionally, public–private partnerships in skill development and agricultural support can further boost the adaptive capacity of vulnerable farming communities.

When assessing social vulnerability to climate change, it is important to consider the relative vulnerability and severity of distribution within the population. The vulnerability measures in the study reveal deviations from the threshold within the vulnerable category in most villages of district Kupwara. The vulnerability gap indicates a shortfall in adaptive capacity, whether in governmental or nongovernmental services and reflects how much an individual or community is exposed to the risk of becoming more vulnerable. The size of the gap also signifies the severity of the vulnerability, as described by Adger^58^, which involves weighing the distribution of the vulnerability gap within the vulnerable population. The blocks predominantly had moderate vulnerability villages, indicating a relatively consistent but still significant level of risk. The presence of a vulnerability gap across blocks highlights the unequal distribution of risk, necessitating differentiated adaptation strategies. In areas with severe vulnerability, interventions must prioritize infrastructure development, economic diversification, and institutional support, whereas in moderate-risk regions, reinforcing existing resilience measures can prevent further escalation of vulnerability. The findings reveal a clear relationship between vulnerability gap, severity, and the distribution of villages across different vulnerability categories within each block. These insights can aid in formulating targeted measures to address specific vulnerabilities and reduce overall risks in the district. Data temporal reliability was upheld primarily by utilizing information from the 2011 census, except financial capacity indicators, which were unavailable. While a composite index for inherent vulnerability was computed using a combination of indicators, any temporal disparities in these two indicators would have a negligible effect on the study’s outcomes. The generated composite index of inherent vulnerability highlighted significant differences across villages and blocks, underscoring the need for context-specific and differentiated adaptation planning efforts in Kupwara. Strengthening the local economy, promoting sustainable land management, and enhancing food security are imperative measures^1,59^ to effectively respond to inherent vulnerability conditions. Reducing inherent vulnerability in the Himalayan region requires a comprehensive approach that integrates sectoral and cross-sectoral interventions. Sectoral initiatives such as National Adaptation Fund for Climate Change (NAFCC) and Pradhan Mantri Krishi Sinchayee Yojana (PMKSY) enhance climate resilience in agriculture, while Jal Jeevan Mission (JJM) and Atal Mission for Rejuvenation and Urban Transformation (AMRUT) strengthen water security and infrastructure. Health programs like National Health Mission (NHM) improve preparedness for climate-sensitive diseases. Cross-sectoral efforts, including Mahatma Gandhi National Rural Employment Guarantee Act (MGNREGA), promote livelihood security and environmental sustainability, while State Action Plans on Climate Change (SAPCCs) and National Disaster Management Plan (NDMP) ensure a coordinated approach to climate adaptation and disaster resilience. Effective implementation, community engagement, training programmes on climate adaptation and policy integration are crucial for building long-term resilience in the region.

## Conclusion

This study highlights that a significant proportion of villages in the Kupwara district exhibit moderate to very high vulnerability, emphasizing the urgent need for targeted interventions. Addressing this challenge requires improving infrastructure, strengthening local institutions, promoting diverse livelihood options, and enhancing market access to build adaptive capacity within rural communities. Given the complex interplay of socioeconomic and environmental factors, an indicator-based vulnerability index was the most effective approach, allowing for a systematic, data-driven assessment across multiple dimensions. The current study, which employed an indicator-based approach using secondary data, faced several limitations, primarily related to data availability and indicator selection. The absence of some critical factors may have influenced the accuracy and comprehensiveness of the vulnerability assessment. A more comprehensive dataset with additional indicators would have allowed for a more nuanced and holistic assessment of inherent vulnerability, providing deeper insights into the complex challenges faced by agricultural communities in Kupwara district. Nonetheless, this assessment is constrained by certain limitations, including data availability, the spatial and temporal resolution of indicators, and assumptions made during the selection and normalization processes—all of which introduce some degree of uncertainty into the results. These uncertainties may influence the precision of vulnerability estimates and affect the prioritization of high-risk areas. Specific sources of potential bias include reliance on the 2011 Census data, temporal mismatches with more recent thematic layers, and the imputation of missing values—particularly for indicators with more than 5% missing data. Although missing data were generally minimal (less than 3% per indicator), the normalization and PCA-based weighting processes may amplify or dampen uncertainties at the indicator level. We acknowledge these limitations and recommend the application of robust sensitivity analyses (e.g., Monte Carlo simulation) in future studies to assess the influence of these uncertainties. Despite these challenges, the adopted methodology offers a transparent and replicable framework for identifying vulnerable regions and guiding informed policy interventions. The overall vulnerability rankings remain robust, with an estimated variation within ± 0.02 index units. Moving forward, efforts to integrate real-time and high-resolution data, expand the range of relevant socio-economic and environmental indicators, and adopt participatory approaches involving local stakeholders will be critical to reducing uncertainty, improving the robustness of vulnerability assessments, and developing resilient, community-driven strategies for sustainable agricultural livelihoods in the region.

## Data Availability

The data that support the findings of this study are available on request from the corresponding author upon reasonable request.
